# Isolation of B Cells Using Silane-Coated Magnetic Nanoparticles

**DOI:** 10.1155/2024/8286525

**Published:** 2024-10-30

**Authors:** Amir Hossein Haghighi, Abolfazl Ghaderian, Esmaeil Mirzaei

**Affiliations:** ^1^Department of Polymer Engineering, Islamic Azad University, Shiraz Branch, Shiraz, Iran; ^2^Young Researchers and Elite Club, Islamic Azad University, Shiraz Branch, Shiraz, Iran; ^3^Department of Medical Nanotechnology, School of Advanced Medical Sciences and Technologies, Shiraz University of Medical Sciences, Shiraz, Iran

**Keywords:** B cell isolation, FITC anti-human CD20 antibody, magnetic nanoparticles, silane

## Abstract

One of the most important advantages and applications of coated nanoparticles in biological applications is their use in isolating different types of cells to diagnose and treat all types of diseases. Therefore, in this research work, the possibility of isolation and enrichment of B cells using magnetic iron oxide nanoparticles have been investigated. In this regard, magnetic nanoparticles are first coated with (3-aminopropyl)triethoxysilane to make them hydrophilic and prevent their clumping, then reacted with and rendered biocompatible by FITC anti-human CD20 antibody. These nanoparticles containing antibodies have been used to isolate B cells from the lymphatic cells. Transmission electron microscopy (TEM) and vibrating-sample magnetometry (VSM) tests were used to check the magnetic properties and coating of nanoparticles. The flow cytometry and fluorescent microscopy tests are used to check antibody binding to nanoparticles. Moreover, flow cytometry tests were used to check the extent of cell separation. Results show that nanoparticles reacted with 450 *μ*L of antibody (T450) performed better than other nanoparticles in isolating B cells.

## 1. Introduction

Today, nanoparticles are used in many applications such as separation of metal ion and other materials [1–3], sensors [4, 5], improving the properties of polymers [6–8], and energy [9, 10] [11–13], and magnetic nanoparticles (MNPs) have a special application in all sciences, especially in medicine, due to their magnetic properties and the possibility of separating and controlling them by an external magnetic field [14–16]. The use of MNPs in medical applications has received tremendous attention, such as the isolation of different types of cells [17]. Cell isolation is utilized in many fields, including the diagnosis of diseases such as cancer and AIDS [18–23]. To put it another way, they can be used to produce a variety of diagnostic kits. Due to their unique properties, such as superparamagnetic and supersaturation, as well as their required size and suitable physical and chemical properties and their high biocompatibility, MNPs have attracted the attention of researchers [24, 25]. These nanoparticles have been reported in varying amounts down to a size of less than 10 nm. This value is important because considering the dimensions of the cell (10 to 100 *μ*m), the virus (20 to 450 nm), the gene (10 nm wide and −1 to 100 nm long), and the protein (3 to 50 nm), it enables penetration and even labeling of all types of cells [26–28]. On the other hand, through the use of various surface coatings, it is possible to create favorable and stable biomedical properties for these MNPs and to prevent their toxicity through the interactions with cells or biological proteins, which leads to an increase in the biocompatibility of MNPs [29–32]. In addition, using modified MNPs does not require the use of centrifuges compared to existing methods in medicine; it also reduces the time and costs of testing because nanoparticles may be isolated through an external magnetic field [33]. Therefore, the cells binding to the surface of MNPs by a biological agent, such as proteins, peptides, and polysulfides, can be easily isolated from blood or another environment [34–37].

The human CD20 antigen is a 35-KD transmembrane glycoprotein found on the surface of pre- and mature B lymphocytes. Furthermore, this antigen is expressed in more than 99% of B-cell non-Hodgkin's lymphomas [38]. In addition, the human CD19 antigen is a 95-KD transmembrane glycoprotein found on the surface of normal and neoplastic B lymphocytes and follicular dendritic cells. The CD19 gene involves B cells' development, proliferation, and differentiation. CD19 forms a multimolecular complex on the surface of mature B cells with CD21 (CR2) and CD81 (TAPA-1) as well as CD225 as a dominant signaling component. Also, CD19 plays two main roles in human B-cell activation: First, it regulates the recruitment of cytoplasmic signaling proteins to the membrane. Second, in the complex, it regulates the B-cell receptor signaling pathways [39–41]. Because these two antigens (CD19 and CD20) can be seen on the surface of almost all B cells, in this study, the anti-human CD20 antibody is used to bind to the modified nanoparticle with silane for the separation of B cell and anti-human CD19 antibody is used for confirmation of isolated B cell in the flow cytometry test.

Immunological isolation has been used in various scientific fields, such as molecular biology, microbiology, and immunology. The use of MNPs constitutes one of the isolation methods, described below in some of the experiments conducted to isolate B cells. In order to isolate the target cell from the environment, nanoparticles must be coupled to a specific antibody or so-called monoclonal antibody so that only the desired cell can be separated from the rest of the cells.

In one of the research types, the CD4+ T lymphocytes were isolated from mouse splenocytes using gold-coated Fe_3_O_4_ nanoparticles. For the isolation, nanoparticles and cells were coupled together with streptavidin–biotin conjugation. The results have shown that compared to the control sample, there are more than 57% greater amounts of CD4+ after isolation [42]. In another study, B and T cells were isolated from PBMC (peripheral blood mononuclear cell) using bacterial magnetic particles (BacMNPs). BacMNPs possess a single magnetic domain of magnetite and can be separated with an external magnetic field. In this regard, they first coupled protein A to BacMNPs and then coupled with anti-mouse IgG antibody. In the following step, PBMCs were incubated with modified BacMNPs and the samples were finally analyzed using flow cytometry. The positive isolation rate of cells expressing CD19 and CD20 markers is more than 98%, indicating BacMNPs' good performance and their ability to isolate these cells from PBMC [43]. In another study, B-type lymphocytes were isolated from blood through 3 stages of purification using the MAXS system and superparamagnetic particles manufactured by Biotech. The isolation mechanism was based on biotin–streptavidin coupling between cells and nanoparticles. For this purpose, PBMCs or lymphocyte cells were first isolated from the red blood cells by FicoLEX, and in the following step, T cells were isolated using the CD3 monoclonal antibody (isolation in the form of negative enrichment). In the final step, using CD20 antibody, they isolated type B cells (isolation by positive enrichment), with the isolation rate more than 97% [44]. In another study, the magnetic-activated cell sorting (MACS) bead isolation kits made by the Miltenyi were used for the isolation of B cells. In this study, three negative isolation or selection kits (separation of other cells) and one positive selection kit (isolation of B cells) were used. The results showed that there are impurities in all the kits and the separation efficiency was between 60% and 99% [45]. Also, in different research types, they used nylon wool column to isolate B cells. After separating the B cells with a centrifuge from tonsillar material, they isolated the B cells with the nylon wool and compared to the kits of antibody-coated magnetic beads (MACS beads). They stated that they can isolated all B-cell subsets, while MACS can isolated certain B-cell subtype and also preserves proliferative and differentiation capacity of the B cells in this method [46]. As can be seen, little work has been done to isolate B cells positively. Therefore, according to the importance of B cells in treating diseases such as cancer and also according to the expression of CD19 and CD20 markers on almost all types of B cells, the purpose of this research is to investigate the possibility of isolating and enriching these cells using magnetite–iron oxide nanoparticle that modified with anti-human CD20 antibody.

## 2. Materials and Methods

### 2.1. Materials

Magnetic iron oxide nanoparticles (Fe_3_O_4_) were obtained from the US company Research Nanomaterials with a polyvinylpyrrolidone (PVP) coating of 1% and a purity of more than 98%. 3-Aminopropyltrimethoxysilane (Silane) of 97% purity, N-hydroxysuccinimide (NHS) of 98% purity, and N-(3-aminopropyl)-N-ethylcarbodiimide hydrochloride (EDC) were purchased from Sigma-Aldrich. The PE anti-human CD19 antibody was bought from BioLegend Company, with a 200 *μ*g/mL concentration. FITC anti-human CD20 antibody with a concentration of 0.6 mg/mL was purchased from Roshd Company. Also, phosphate-buffered saline (PBS 1x) was used as a buffer solution.

### 2.2. Tests

At room temperature and with a field strength of 10 kOe, a vibrating-sample magnetometer (VSM) from Magnetic Daneshpajouh Kashan (MDK) was used to identify the magnetic properties of nanoparticles and to investigate the coating of nanoparticles with silane. Transmission electron microscopy (TEM) testing has been performed in a Philips EM208S 100 KV device to establish the coverage of nanoparticles with APTES. X-ray diffraction (XRD) was used to obtain the crystallography pattern of the MNPs. The data were taken from a Bruker AXS D8 ADVANCE X-ray diffractometer (Cu K*α* radiation with *λ* = 1.540 Å) at 25°C. In order to check the antibody coupling to the nanoparticles, identify the nanoparticles coupled to the target cell, and isolate them from the cellular environment, fluorescent microscope tests and flow cytometry were used. A BD FACSCalibur device was used for flow cytometry testing, and a BX61 Olympus machine was used for the fluorescent microscope.

### 2.3. Methods

#### 2.3.1. Method of Coating Nanoparticles With Silane

The coating of nanoparticles with silane was carried out by the method described by Abu Reziq et al. [47], which was fully described in our previous study [19], and the various tests performed to check the coating of MNP with silane were mentioned in this article. In this study, two important tests include TEM and VSM have been presented to show the silane coating on the surface of MNP and to check the magnetic property.

#### 2.3.2. Antibody Coupling Method to Silane-Coated Nanoparticles

As shown in Table 1, a certain amount of antibody with a concentration of 0.6 mg/mL is poured into a 2 mL Eppendorf tube in the first step. In the second step, in order to activate the antibody, EDC and NHS were weighed and dissolved in 20 to 30 *μ*L of distilled water and then added to the tube containing the antibody, and vortexed. They were placed on the shaker for 30 to 40 min to be fully activated. In order to protect the dye of FITC from light, tubes were covered with aluminum foil. In the third step, 350 *μ*L nanoparticles with a 3 mg/mL concentration were sonicated and added to the tube containing the activated antibody. It was placed on a tubular shaker for 4 h for antibody binding to MNP. It should be noted that the aluminum cover was placed over the tubes during the entire reaction. Finally, the nanoparticles were washed, and unreacted antibody and EDC/NHS were removed from the MNPs containing the antibody (MNP-Ab).

**Figure 1:**
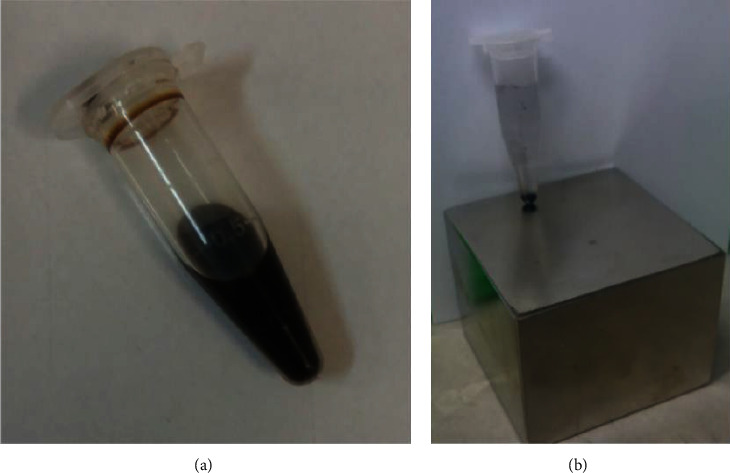
(a) Image of sonicated magnetic nanoparticles and (b) deposition of nanoparticles placed on the magnet.

The MNP-Ab was washed with PBS 1x in three steps and stored in a storage buffer. As shown in Figure 1, we used magnets and not centrifuge devices for washing. 1 mL PBS 1x was added to the tube with reaction materials, and the tube was put on the magnet for 10 min in order to completely deposit the nanoparticles, and then, the supernatant was discarded. The second and third steps are the same. Finally, 1 mL of storage buffer was added to MNP-Ab, vortexed, and placed them in the refrigerator. Note that the storage buffer contains 99.9% PBS 1x, 0.1% FBS, and 0.05 g of sodium azide.

#### 2.3.3. Checking Antibody Binding to Nanoparticles

To study the coupling of FITC anti-human CD20 antibody to silane-coated MNPs, these MNP-Abs were first sonicated at a concentration of 1 mg/mL in an ice bath at low voltage (between 10 and 20 kHz) and pulsed (10 s off and 10 s on) until fully homogenized. Then, according to Table 2, a 30 *μ*L of nanoparticles was added to the flow cytometric tubes. Then, 250 to 350 *μ*L of PBS 1x was added to all the tubes, vortexed, and analyzed by flow cytometry. For comparison, MNPs without antibodies have been considered as control samples. All nanoparticles produced with different amounts of antibody were examined similarly.

#### 2.3.4. The Method of PBMC Isolation From Blood

To isolate lymphocytes or mononuclear cells (PBMCs) from the blood, first, blood is collected from the donor. The lymph is separated with Ficoll and washed with culture medium. Finally, it is washed again with PBS 1x, and the cells or lymph is counted with trypan blue and used based on the required number.

#### 2.3.5. The Method of Target Cell Isolation Using Nanoparticles Containing Antibodies

To investigate the ability of MNP-Ab in separation of target cell, first PBMC or lymphocytes are isolated from blood. The amount of lymph is usually between 1 and 1.5 million. It should be noted that they are sonicated in an ice bath before MNP-Ab, with a concentration of 1 mg/mL, is added to the lymph. Next, a mixture of MNP-Ab and cells was placed on the shaker for 2 h and allowed to bind between MNP-Ab and PBMCs containing the CD20 marker. The unreacted cells are washed out of the reaction medium in the next step. The washing with PBS 1× was done in three steps. During each step, 1 mL PBS 1× was added to each tube, vortexed, and placed onto a magnet for 10 min in order to completely deposit the nanoparticles, and then, the supernatant is discarded. Two more washings are performed. Finally, 200 *μ*L of PBS 1× was added to the mixture, and it was transferred to flow cytometry tubes and stained. To staining all tubes (test and control), 10 *μ*L of PE anti-human CD19 antibody was added to tubes, vortexed, and placed in the dark at room temperature for 15–20 min. After this period, 2 mL of PBS 1× was added to the tubes, and they were washed by centrifugation at 650 × g for 5 min. Then, 300 *μ*L of PBS 1× was added to each tube and analyzed by flow cytometry device.

According to Table 3, in each reaction, a tube entirely similar to the test tube with the same number of cells and MNP-Ab was prepared and used as a control tube to analyze the results. The only difference between the control and the test tube was that the control sample was not placed on a magnet to deposit MNP-Ab; in other words, it was not washed but was stained with PE anti-human CD19 antibody.

## 3. Results

### 3.1. VSM Test of Pure and Coated Nanoparticles

**Figure 2:**
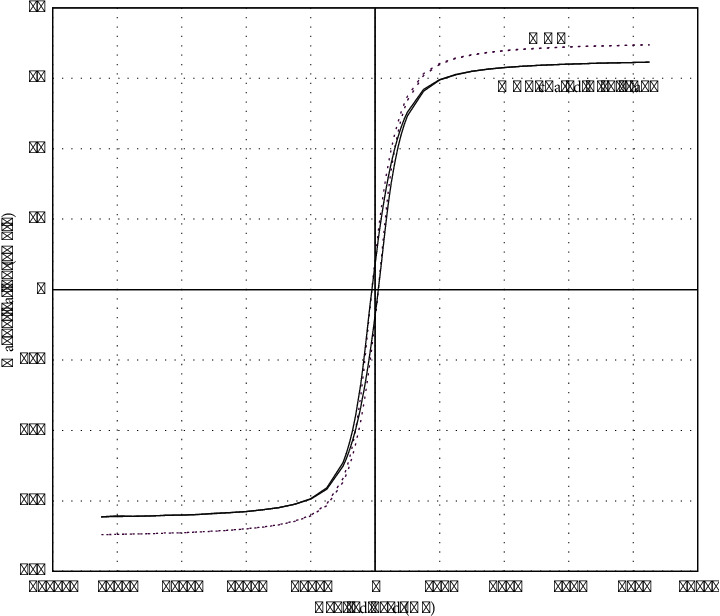
Measurement of magnetic properties of silane-coated and silane-free MNPs using VSM test.

The VSM test at room temperature has been used to investigate the magnetic properties of the nanoparticles and their changes with the applied coating. As can be seen in Figure 2, nanoparticles have paramagnetic properties and are close to superparamagnetic, because the hysteresis curve is very small. In addition, according to Figure 1, sonicated nanoparticles are completely dissolved in water and the color of their solution is black. However, they precipitate quickly and will be immediately colorless when exposed to the magnet or external magnetic field. In addition, according to the studies, the smaller the squareness ratio or Mr/Ms, the greater the magnetic property, and a material whose squareness ratio is zero is superparamagnetic. The squareness ratio of coated nanoparticles is low, as shown in Table 4 [48, 49]. As shown in Table 4 and Figure 2, the magnetic properties of particles appear to have changed slightly when subjected to a coating, so that the saturation magnetization value of silane-coated MNP compared to pure nanoparticle has decreased by about 5 emu/gr and changed from 69.5 to 64.5. The reason for this reduction in saturation magnetism can be explained by the presence of a nonmagnetic layer of silane on the surface of the nanoparticles, and this change itself indicates the coating reaction and the creation of a layer of silane on the surface of the nanoparticles [49, 50].

### 3.2. TEM Test of Coated Nanoparticles

**Figure 3:**
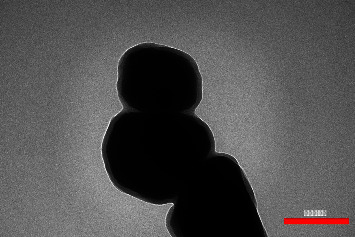
TEM image of magnetic nanoparticles coated with silane.

To observe the coating of nanoparticles due to the fact that the coating on the surface of nanoparticles was not well defined in scanning electron microscopy (SEM) images, the TEM test was used to observe the coating of nanoparticles. As shown in Figure 3, (3-aminopropyl)triethoxysilane covers the surface of the MNPs well, thus preventing them from adhesion and forming clumps. In addition, the thickness of the silane coating on the surface of the nanoparticles was measured with the software and it was found to be 15 ± 3 nm on average. Also, the previous study includes the SEM images of nanoparticles with and without silane [19].

### 3.3. XRD Test of Nanoparticles

**Figure 4:**
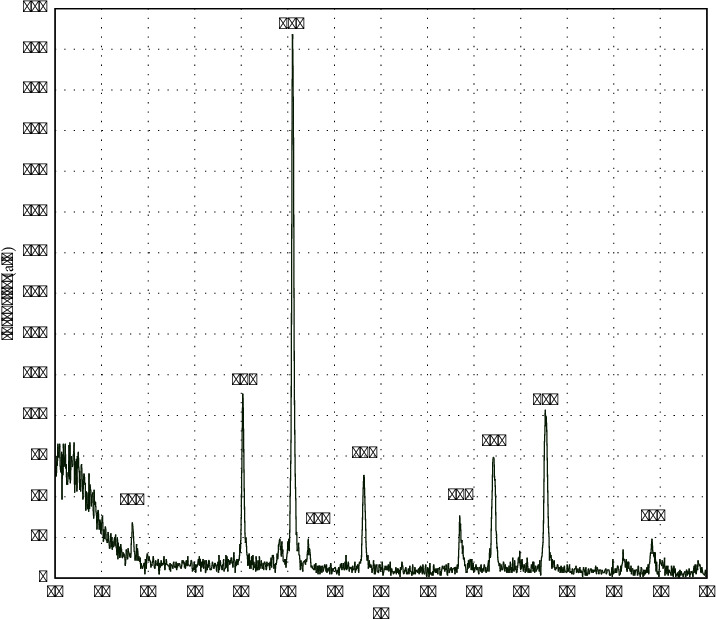
XRD patterns of magnetic nanoparticles.

The crystalline structure of the MNPs was investigated with the XRD test. The peaks in Figure 4 related to crystalline pages 111, 220, 311, 222, 400, 422, 511, 440, and 533, which represent the inverse spinel structure of Fe_3_O_4_ [51, 52]. According to previous studies, applying a coating on the surface of nanoparticles did not change their crystal structure and only created a broad peak in the range of 2*θ* equal to 10–20 [19, 53].

### 3.4. Conjugation of Anti-Human CD20 Antibody on the Surface of Coated Nanoparticles

There are two methods or strategies to couple antibodies or immobilize them on the surface of nanoparticles: nonoriented binding and oriented binding. In nonoriented binding, the antibody is placed directly on the surface of the nanoparticles, and their coupling to each other is generally of the type of ionic-based interactions. This type of interaction decreases the efficiency of the antibody or reduces the active sites for coupling the antibody to the antigen due to steric hindrance or coupling of the antibody to the nanoparticles via the active sites in the Fab (paratube) of the antibody. Nevertheless, the antibody is connected to the nanoparticle's surface through a covalent bond in the targeted or oriented binding. Since this connection is from the Fc region of the antibody, the efficiency of the antibody does not change and increases compared to the previous state, which is caused by the remaining active sites. In order to achieve the best possible antibody efficacy, binding should be performed from the Fc region of the antibody to the surface of the nanoparticles [35, 54, 55]. Furthermore, the determining factor in the isolation of cells is the antibody and its correct binding to nanoparticles. The main role of SiO_2_ is biocompatibility and preventing the destruction of iron oxide nanoparticles, as well as strong (chemical binding) and correct (from the area of Fc antibody) binding of antibodies to it, and for this reason, the effect of different coatings and their degree of biocompatibility have been investigated in various research types [17, 56–58]. Also, further increase of SiO_2_ only increases the thickness of the coating and further decreases the magnetic property of nanoparticles, because according to Figure 2, the magnetic property decreases with the SiO_2_ coating.

The objective of this research work is to covalently couple nanoparticles to antibodies. In most of the research works for coupling antibodies to nanoparticles and according to the nature of the functional groups on their nanoparticle surface, the nanoparticles were first activated with an active substance and then reacted with the antibody [59–61] in some research works the process is reversed. First, the antibody was activated with an activator, and then, nanoparticles were added to it [62, 63]. One of these activators is EDC/NHS, used for compounds with an acidic functional group. In the studies where the nanoparticles are coated with an acidic group, they are activated with EDC/NHS and then treated with antibodies to react with the amino group of antibodies to form an amide bond [42, 61, 64]. However, this reaction path reduces the efficiency of the antibody and, as a consequence, reduces the coupling to the antigen of the target cell because the activated acid usually reacts with the primary amine, and this amine is located at the specific site of the antibody to the antigen or Fab region of the antibody [35].

In this work, 3-aminopropyl trimethoxy silane was used to cover the nanoparticles, so these nanoparticles with a positive surface charge and an amine functional group oppositely react with the antibody, that is, first the antibody is activated by EDC/NHS, and then, the nanoparticles with the amino group are added to them. One of the advantages of this method is that it is an easy and fast way to react between nanoparticles and antibodies without the need for isolation during the reaction and after activation with EDC/NHS, and the isolation only occurs at the end of the reaction. The results from flow cytometry and fluorescent microscope tests confirm the coupling of antibodies and nanoparticles to each other. The results of which will be explained below.

#### 3.4.1. Investigating Antibody Coupling to Nanoparticles Using Flow Cytometry Technique

Flow cytometry tests are used to detect antibodies that couple to nanoparticles. Unlike many other studies, which are limited to the expression of antibodies coupling to nanoparticles [47, 59, 65], or based on the amount of absorption, the amount of antibody has been determined in a relative manner [66]. In addition to considering the conjugation of antibodies to nanoparticles, the results of this test also numerically express a measure of the amount of binding. Therefore, in the flow cytometry test, if antibodies are conjugated to nanoparticles, a difference in mean fluorescence intensity (MFI) is observed between nanoparticles without and with antibodies due to the FITC dye coupled to the antibody; the nanoparticle with antibody has a higher MFI in compare nanoparticle without antibody. It should be noted that the antibody used in this study, i.e., FITC anti-human CD20 antibody has a FITC dye, can be easily distinguished from the nanoparticles without antibodies on the flow cytometry test, and does not need to be stained. Suppose an antibody does not have any dye. In that case, it should be stained first with a fluorescent dye or antibody has a fluorescent dye, and then analyzed by flow cytometry, as in a previous study for cancer cell separation [19].

**Figure 5:**
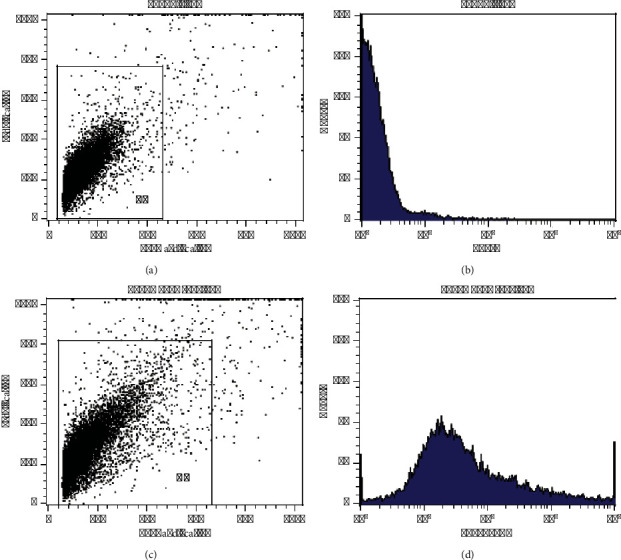
Flow cytometry diagram of the sample of T450 (graphs (c) and (d)) along with the control sample or nanoparticle without antibody (graphs (a) and (b)).

For the analysis of a sample with flow cytometric, as shown in Figure 5, first obtain a side scatter versus a forward scatter diagram, and then, according to the abundance of nanoparticles and their position, select or gate the nanoparticles, and then obtain a graph of changes in color intensity and compared it to the control sample. For instance, a diagram of the sample T450 and its control graph can be observed in Figure 5. As can be seen, the color intensity change graph for the T450 sample (Figure 5(d)) is completely different from the control sample (Figure 5(b)), and this shows that the antibody is conjugated to the nanoparticles. These changes are expressed by the MFI parameter, which is less than 5 in the control sample containing the nanoparticles without antibodies and is approximately 2 ± 1 according to the calculations of the device, but in the sample T450, it is 42 ± 2. This increase in MFIs indicates that an antibody is conjugated to the surface of nanoparticles.

#### 3.4.2. Investigating the Effect of the Amount of Antibody on Its Coupling to Silane-Coated Nanoparticles

To investigate the effect of the antibody on its binding to nanoparticles and determine the optimal amount of antibody, reactions with different amounts of antibody at a constant concentration of nanoparticles (3 mg/mL) were performed. In addition, due to the importance of the reaction volume in the interaction between antibody and nanoparticles, the amount and concentration of materials were chosen in such a way that the minimum possible volume is obtained and more antibodies are attached to the surface of nanoparticles; for this reason, the volume of reaction is mentioned in Table 1. In most articles, the main goal was to couple the antibody on the surface of the nanoparticles. For this reason, the effect of the amount of antibodies on the surface of the nanoparticles is not reported in various articles. In one of the research works, 4 mg of Herceptin antibody was used to react with 0.2 mg of nanoparticles [67]. However, this problem was studied and analyzed with different and smaller amounts of antibodies in reaction with nanoparticles to obtain an optimal and desirable amount. As can be seen in Table 5, the antibody reaction with silane-coated MNPs used a maximum of 0.27 mg and a minimum of 0.015 mg of antibody for the reaction, which is lower than the values reported in many references, and because of the very high cost of antibodies, the fluorescent color intensity parameter (MFI) and the reaction conditions are very useful for reducing the cost reduction and achieving the desired result [59, 61, 62].

**Figure 6:**
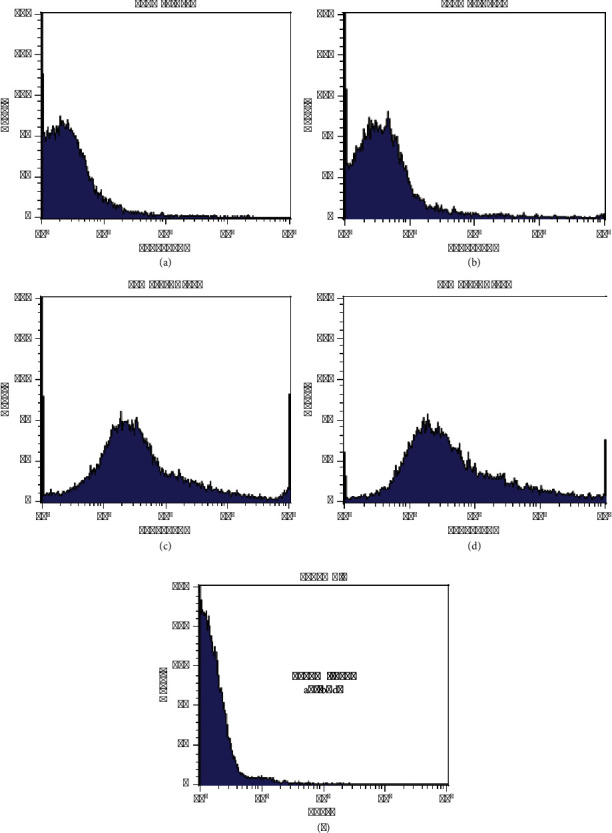
Histogram plot of coupling of silane-coated MNPs with FITC anti-human CD20 antibody at four different amounts of antibody (Figures (a), (b), (c), (d), and (e) correspond to T25, T100, T300, T450, and MNPs without antibody, respectively).

As can be seen from the flow cytometry histogram plots in Figure 6, as the amount of antibodies decreased, the amount of their binding to nanoparticles and also MFI decreased. These changes are evident from the fluorescence intensity or moving the blue graph in Figure 6 to lower amounts of CD20-FITC compared to T450 and near nanoparticles without antibodies. The MFI and results in different reactions are shown in Table 5; the highest MFI value is 42, which refers to T450 (Figure 6(d)), utilizing the highest amount of antibodies in its production, and the lowest amount of MFI is 4, which refers to the T25 sample (Figure 6(a)), which used the lowest amount of antibodies in its synthesis.

**Figure 7:**
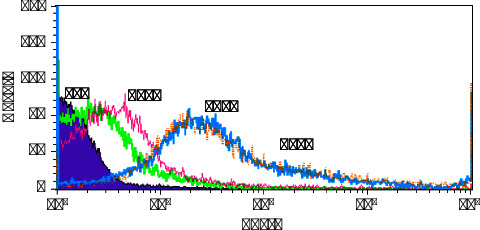
Histogram diagram of the reaction of nanoparticles with different amounts of antibody in one graph for comparison (purple graph nanoparticles without antibody).

As can be seen from the flow cytometry histogram graphs in Figures 6 and 7, with the decrease in the amount of antibodies, the amount of their binding to nanoparticles has also decreased, and these changes can be seen from the MFI.

In Figure 7, all samples are placed together in one diagram for comparison. Conjugation in all samples is done, and the graph of nanoparticles containing antibodies is placed ahead of the purple graph (the graph of nanoparticles without antibodies or control samples), which shows the binding of antibodies to nanoparticles even in tiny amounts of antibodies (sample T25 with 0.015 mg of antibody). In addition, Table 5 states a column of the weight ratio of nanoparticle to antibody; this ratio has increased from 4 to 70 times. The results show that the antibody is binding to the nanoparticle, even when the weight ratio of the nanoparticle to the antibody is increased to 70 times, which confirms that the reaction is done correctly. The coupling levels were very low in the T25 and T100 samples; for this reason, they have not been used in cell isolation, but T300 and T450 have more significant amounts of antibodies to have been used for cell separation.

As can be seen in Figure 7, in two samples, T300 and T450, MFI and coupling of antibodies to nanoparticles are almost the same, and increasing the amount of antibodies is not logical because further increase can reduce the efficiency of MNP-Ab.

#### 3.4.3. Examining Antibody Coupling to Nanoparticles Using a Fluorescent Microscope

**Figure 8:**
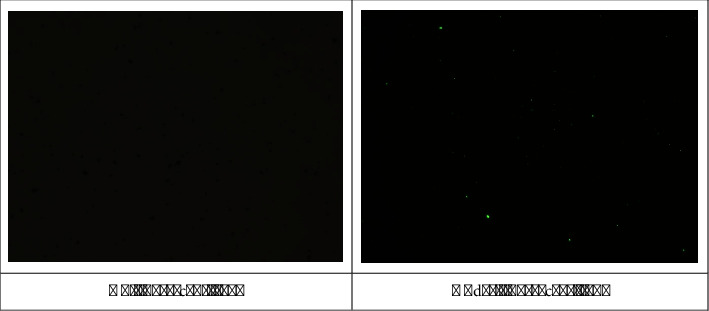
Fluorescent microscopy images of nanoparticles containing antibodies.

Fluorescence microscopy is another method for detecting antibody coupling to nanoparticles because the antibody coupled to the nanoparticles (FITC anti-human CD20) has the FITC dye (fluorescein isothiocyanate), which appears green under the fluorescence microscope and at the corresponding wavelength (excitation and emission wavelengths approximately 495 nm and 519 nm). Consequently, the nanoparticles would be seen in green color under a fluorescent microscope if an antibody was coupled to their surface. Accordingly, as seen in Figure 8, the antibody is coupled to the surface of the nanoparticles, so they are seen in green. It is also possible to examine cells using this technique. However, finding them under fluorescent microscopes has been very difficult and time-consuming, given the extremely small number of isolated cells.

### 3.5. Investigating the Coupling of MNP-Ab to Target Cells and Isolation

After binding the antibody to the coated nanoparticles, they were adjacent to the PBMCs and then washed and analyzed using the flow cytometry test after the reaction time. During this phase, the target cells have been isolated using nanoparticles with different MFI values. However, the results showed that the T25 and T100 samples did not possess the required ability to isolate the target cells due to the low amount of antibodies and lacked efficiency. Therefore, for the isolation of B cells, we used MNP-Ab with higher MFI values and therefore have a higher antibody; for this reason, only results and graphs were reported for T300 and T450 nanoparticles.

**Figure 9:**
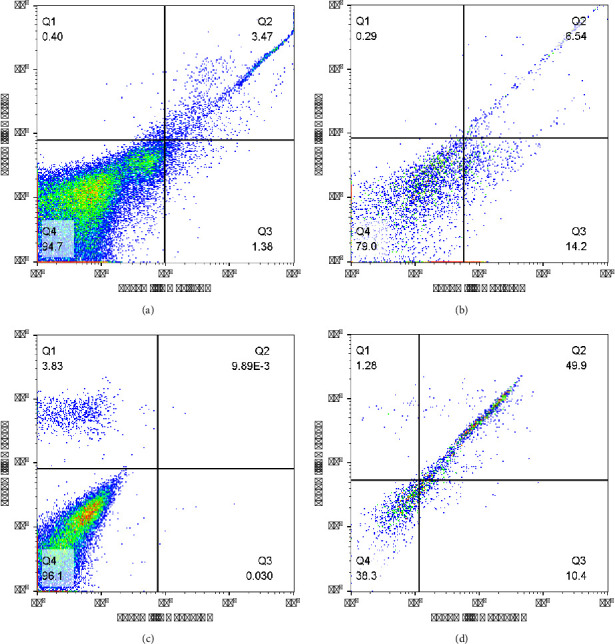
Flow cytometry histogram of B-cell isolation using T300 (graphs (a) and (b)) and T450 (graphs (c) and (d)) nanoparticles (graphs (a) and (c): control sample; (b) and (d): test sample).

Figure 9(a) and 9(b) show that T300 has an efficiency of 47%; the percentage of B cells in the control sample is 3.47% and in the test sample where the cells were isolated and washed is 6.54%. In another test with T450 nanoparticles, which can be seen in Figures 9(c) and 9(d), the percentage of cells in the control sample was 0.09%, which increased to 49.9% after isolation, and the isolation efficiency in this case was 99%, indicating the superior performance of T450 nanoparticles compared to T300. This is because the antibody content of the T450 nanoparticles is higher, which increases the ability of MNP-Ab to couple B cells and isolate them from the environment. However, the major problem in isolating B cells with MNP-Ab was the presence of clumping or adhesion of cells to each other. Unfortunately, when MNP-Ab is added to cells, a large cluster is formed during the reaction or washing, and cells and nanoparticles are coupled to each other and together. It was even washed several times, but the desired result was not achieved. Cluster and coupling of cells and nanoparticles were also observed in the T450 sample, where a higher amount of antibody. The point is that if the cluster is formed and the cells stick together, it is impossible to analyze them with a flow cytometer device. These tests (reaction of cell with MNP-Ab) were done several times, but unfortunately, due to the adhesion cells to each other, analysis by flow cytometry was not done. Even in one of the tests, DNase and Max buffers were used instead of PBS buffer, and the number of lymph cells was reduced to prevent cell adhesion, but cluster was also observed in this test. In another experiment, in order to prevent the death of cells and their coupling to each other, a reaction between cells and nanoparticles was performed at a temperature lower than the ambient temperature in the refrigerator, but the cells adhered to each other. The desired effect was not achieved, even when a combination of different factors, such as the use of DNase and the reaction in the refrigerator or the reduction of lymph, was attempted and the adhesion of the materials to each other occurred. In another experiment, they were sonicated (at low voltage and pulsed) to eliminate cluster formation and adhesion of materials to each other, but good results were not obtained after analyzing them with the flow cytometry test. For this reason, the isolation rate of cells was low, and according to the obtained results, it seems that MNP-Ab coated with silane does not have the necessary and sufficient ability to isolate B cells. It should be mentioned that in another study, antibodies binding to nanoparticles coated with silane were used for the isolation of breast cancer cells, in which the cancer cells were isolated from the blood environment and PBMCs, and the efficiency and performance of the nanoparticles in isolation were excellent and favorable [19, 21]. However, in this study, there was a problem with cell adhesion, and this difference in performance could be caused by the different structures of antibodies and the type of cells.

## 4. Conclusion

The results of TEM and VSM tests showed that silane covered the surface of the nanoparticles, and the magnetic property was reduced to a very small extent by using silane coating (from 69.5 to 64.5 emu/gr). Flow cytometry confirmed the binding of FITC anti-human CD20 antibody in all four different amounts of antibody (25, 100, 300, and 450 *μ*L) to the surface of nanoparticles coated with silane, but the amount of binding in the samples of 300 and 450 *μ*L of antibody compared to the samples of 25 and 100 *μ*L was significantly higher. Their MFI values are equal to 36 and 42, respectively. In addition to the flow cytometry test, the color change observed under the fluorescent microscope caused by nanoparticles containing antibodies compared to nanoparticles without antibodies due to the presence of FITC dye attached to the antibody confirms the binding of the antibody to the surface of the nanoparticles. T300 and T450 were used to isolate B cells. Experiments showed that T300 and T450 nanoparticles have efficiencies of 47% and 99% in isolating CD19 cells, respectively. As a result, T450 nanoparticles have better performance than T300 nanoparticles and other nanoparticles in isolating cells due to the lower adhesion of cells to each other in the separation process and also have a higher antibody.

## Figures and Tables

**Table 1:** Amount and type of materials used in the reaction between silane-coated MNPs and antibody (amount of MNPs (350 µL with 3 mg/mL concentration), amount of EDC (8 ± 2 mg), amount of NHS (14 ± 2), reaction time (4 h)).

**Test**	**Amount of CD20 antibody (μL)**	**Amount of MNP (mg)**	**Reaction volume (mL)**
1	25	1	0.500
2	100	1	0.550
3	300	1	0.750
4	450	1	0.950

Abbreviation: MNP, magnetic nanoparticles.

**Table 2:** Amount and type of materials to check antibody coupling to nanoparticles by flow cytometry.

**Nanoparticle type**	**Flow cytometry tube**	**Amount of nanoparticles (*μ*L)**
MNP-Ab	1	30
Silane-coated MNPs without antibody	2	30

**Table 3:** Characteristics of test and control tubes in the reaction between PBMCs and MNP-containing antibodies (amount of PBMCs in both tubes are equal).

**Tube**	**Washing**	**Staining with PE anti-human CD19**	**The amount of MNP-Ab (1 mg/mL) (*μ*L)**	**Amount of PBMCs (million)**	**Reaction volume (*μ*L)**
Test	Yes	Yes	15–20	1–1.5	120 ± 5
Control	No	Yes	15–20	1–1.5	120 ± 5

Abbreviations: MNP, mcagnetic nanoparticles; PBMC, peripheral blood mononuclear cell.

**Table 4:** The results of the VSM test of nanoparticles.

**Nanoparticle type**	**Saturation magnetization value (Ms) (emu/gr)**	**Magnetic residue value (Mr) (emu/gr)**	**Magnetic reluctance value (Hc) (Oe)**	**Squareness ratio (Mr/Ms)**
Pure nanoparticle	69.5	8.3	98	0.11
Silane-coated MNPs	64.5	7.05	98	0.11

**Table 5:** The effect of the amount of antibodies on their binding to the nanoparticles.

**Test name**	**Amount of CD20 antibody (*μ*L)**	**Amount of CD20 antibody (mg)**	**MFI**	**Ratio of nanoparticle to antibody**
T25	25	0.015	3	70
T100	100	0.06	5	17
T300	300	0.18	36	6
T450	450	0.27	42	4

Abbreviation: MFI, Mean Fluorescence Intensity.

## Data Availability

Research data are not shared.

## References

[1] Gürbüz M. U., Elmacı G., Ertürk A. S. (2021). In Situ Deposition of Silver Nanoparticles on Polydopamine-Coated Manganese Ferrite Nanoparticles: Synthesis, Characterization, and Application to the Degradation of Organic Dye Pollutants as an Efficient Magnetically Recyclable Nanocatalyst. *Applied Organometallic Chemistry*.

[2] Kayili H. M., Ertürk A. S., Elmacı G., Salih B. (2019). Poly(amidoamine) Dendrimer-Coated Magnetic Nanoparticles for the Fast Purification and Selective Enrichment of Glycopeptides and Glycans. *Journal of Separation Science*.

[3] Li S., Li X., Li S., Xu P., Liu Z., Yu S. (2024). In-Situ Preparation of Lignin/Fe_3_O_4_ Magnetic Spheres as Bifunctional Material for the Efficient Removal of Metal Ions and Methylene Blue. *International Journal of Biological Macromolecules*.

[4] babaei Z., Rezaei B., Gholami E., Afshar Taromi F., Haghighi A. H. (2024). In Situ Synthesis of Long Tubular Water-Dispersible Polyaniline With Core/shell Gold and Silver@graphene Oxide Nanoparticles for Gas Sensor Application. *Heliyon*.

[5] Ghosh T., Raj G. B., Dash K. K. (2022). A Comprehensive Review on Nanotechnology Based Sensors for Monitoring Quality and Shelf Life of Food Products. *Measurement: Food.*.

[6] Yaldagard M., Yaldagard J. (2023). A Review of the Effect of Nanoparticles on the Improvement of Mechanical and Dielectric Properties of Polyvinyl Chloride, Nanodielectric Polymer, in Wire and Cable Insulation. *Iranian Chemical Engineering Journal*.

[7] Tsou C. H., Du J. H., Yao W. H. (2023). Improving Mechanical and Barrier Properties of Antibacterial Poly(Phenylene Sulfide) Nanocomposites Reinforced With Nano Zinc Oxide-Decorated Graphene. *Polymers*.

[8] Jiang Z., Cai H., Chen X (2022). Improving the Mechanical and Damping Properties of Polymer/Memory Alloy Composite by Introducing Nanotubes Covered With Nano-Scale Ni Particles. *Composites Part A: Applied Science and Manufacturing*.

[9] Haghighi A. H., Tohidian M., Ghaderian A., Shakeri S. E. (2017). Polyelectrolyte Nanocomposite Membranes Using Surface Modified Nanosilica for Fuel Cell Applications. *Journal of Macromolecular Science, Part B*.

[10] Heiran R., Pashaei B., Sedaghati F., Haghighi A. H., Ghaderian A., Thomas S., Saiter-Fourcin A., Jibin K. P. (2024). 14-Hybrid Nanofillers for Polymer-Based Energy Storage Applications. *Hybrid Nanofillers for Polymer Reinforcement*.

[11] Vijayaram S., Razafindralambo H., Sun Y. Z. (2024). Applications of Green Synthesized Metal Nanoparticles-a Review. *Biological Trace Element Research*.

[12] Zhou X. Q., Hayat Z., Zhang D. D. (2023). Zinc Oxide Nanoparticles: Synthesis, Characterization, Modification, and Applications in Food and Agriculture. *Processes*.

[13] Nie P., Zhao Y., Xu H. (2023). Synthesis, Applications, Toxicity and Toxicity Mechanisms of Silver Nanoparticles: A Review. *Ecotoxicology and Environmental Safety*.

[14] Esmaeili Lashkarian E., Ahmadi S., Beigmohammadi F. (2024). Recent Application of Nanomaterials-Based Magnetic Solid Phase Micro-Extraction for Heavy Metals Food Toxicity. *International Journal of Networks and Communications*.

[15] Sondhi S., Sharma R., Kumar A., Kamwar K., Kaur P. S., Inamuddin N., Cruz J., Altalhi T. (2023). Chapter 8 - Biomedical Applications of Magnetic Nanocarriers-a Review. *Green Sustainable Process for Chemical and Environmental Engineering and Science*.

[16] Munir T., Mahmood A., Rasul A., Imran M., Fakhar-e-Alam M. (2023). Biocompatible Polymer Functionalized Magnetic Nanoparticles for Antimicrobial and Anticancer Activities. *Materials Chemistry and Physics*.

[17] Haghighi A. H. (2024). Isolation of B Cells Using Magnetic Nanoparticles and Investigating the Effect of Cell Staining and the Amount of Nanoparticles on Their Isolation. *Iranian Chemical Engineering Journal*.

[18] Guo L., Liu C., Qi M. (2023). Recent Progress of Nanostructure-Based Enrichment of Circulating Tumor Cells and Downstream Analysis. *Lab on a Chip*.

[19] Haghighi A. H., Faghih Z., Khorasani M. T., Farjadian F. (2019). Antibody Conjugated Onto Surface Modified Magnetic Nanoparticles for Separation of HER2+ Breast Cancer Cells. *Journal of Magnetism and Magnetic Materials*.

[20] Dongsar T. T., Dongsar T. S., Abourehab M. A. S., Gupta N., Kesharwani P. (2023). Emerging Application of Magnetic Nanoparticles for Breast Cancer Therapy. *European Polymer Journal*.

[21] Haghighi A. H., Khorasani M. T., Faghih Z., Farjadian F. (2020). Effects of Different Quantities of Antibody Conjugated With Magnetic Nanoparticles on Cell Separation Efficiency. *Heliyon*.

[22] Materón E. M., Miyazaki C. M., Carr O (2021). Magnetic Nanoparticles in Biomedical Applications: A Review. *Applied Surface Science Advances*.

[23] Khizar S., Elkalla E., Zine N., Jaffrezic-Renault N., Errachid A., Elaissari A. (2023). Magnetic Nanoparticles: Multifunctional Tool for Cancer Therapy. *Expert Opinion on Drug Delivery*.

[24] Laghmouchi A., Hoogstraten C., Falkenburg J. H. F., Jedema I. (2020). Long-Term In Vitro Persistence of Magnetic Properties After Magnetic Bead-Based Cell Separation of T Cells. *Scandinavian Journal of Immunology*.

[25] Tian X., Zhang L., Yang M. (2018). Functional Magnetic Hybrid Nanomaterials for Biomedical Diagnosis and Treatment. *Wiley Interdisciplinary Reviews. Nanomedicine and Nanobiotechnology*.

[26] Issa B., Obaidat I. M., Albiss B. A., Haik Y. (2013). Magnetic Nanoparticles: Surface Effects and Properties Related to Biomedicine Applications. *International Journal of Molecular Sciences*.

[27] Khan I., Saeed K., Khan I. (2019). Nanoparticles: Properties, Applications and Toxicities. *Arabian Journal of Chemistry*.

[28] Setia A., Mehata A. K., Vikas M. A. K., Malik A. K., Viswanadh M. K., Muthu M. S. (2023). Theranostic Magnetic Nanoparticles: Synthesis, Properties, Toxicity, and Emerging Trends for Biomedical Applications. *Journal of Drug Delivery Science and Technology*.

[29] Haghighi A. H., Taherinezhad S., Babaei Z. (2022). A Review on the Properties of the Iron Oxide Nanoparticles Coated With Different Materials Used in Biomedical Applications. *Nano World*.

[30] Zhu N., Ji H., Yu P. (2018). Surface Modification of Magnetic Iron Oxide Nanoparticles. *Nanomaterials*.

[31] Nosrati H., Salehiabar M., Attari E., Davaran S., Danafar H., Manjili H. K. (2018). Green and One-Pot Surface Coating of Iron Oxide Magnetic Nanoparticles With Natural Amino Acids and Biocompatibility Investigation. *Applied Organometallic Chemistry*.

[32] Vassallo M., Martella D., Barrera G. (2023). Improvement of Hyperthermia Properties of Iron Oxide Nanoparticles by Surface Coating. *ACS Omega*.

[33] Estelrich J., Escribano E., Queralt J., Busquets M. A. (2015). Iron Oxide Nanoparticles for Magnetically-Guided and Magnetically-Responsive Drug Delivery. *International Journal of Molecular Sciences*.

[34] Wang X., Li B., Li R. (2018). Anti-CD133 Monoclonal Antibody Conjugated Immunomagnetic Nanosensor for Molecular Imaging of Targeted Cancer Stem Cells. *Sensors and Actuators B: Chemical*.

[35] Trang V. T., Tam L. T., Quy N. V. (2018). Preparation and Characterization of Aminosilane-Functionalized Magnetic Antibody Conjugates for Bacterial Recognition and Capture. *IEEE Transactions on Magnetics*.

[36] Robinson H. R., Qi J., Cook E. M (2018). A CD19/CD3 Bispecific Antibody for Effective Immunotherapy of Chronic Lymphocytic Leukemia in the Ibrutinib Era. *Blood*.

[37] Sahoo S. L., Liu C. H., Wu W. C. (2017). Lymphoma Cell Isolation Using Multifunctional Magnetic Nanoparticles: Antibody Conjugation and Characterization. *RSC Advances*.

[38] Payandeh Z., Bahrami A. A., Hoseinpoor R (2019). The Applications of Anti-CD20 Antibodies to Treat Various B Cells Disorders. *Biomedicine & Pharmacotherapy*.

[39] Wang K., Wei G., Liu D. (2012). CD19: A Biomarker for B Cell Development, Lymphoma Diagnosis and Therapy. *Experimental Hematology & Oncology*.

[40] Tedder T. F. (2009). CD19: A Promising B Cell Target for Rheumatoid Arthritis. *Nature Reviews Rheumatology*.

[41] Cano R., Cano R. L. E., Lopera H. D. E. (2013). Chapter 5 Introduction to T and B Lymphocytes. *Juan-Manuel Anaya M, PhD, Yehuda Shoenfeld, MD, FRCP (UK)*.

[42] Liu H. L., Sonn C. H., Wu J. H., Lee K.-M., Kim Y. K. (2008). Synthesis of Streptavidin-FITC-Conjugated Core–Shell Fe_3_O_4_-Au Nanocrystals and Their Application for the Purification of CD4+ Lymphocytes. *Biomaterials*.

[43] Kuhara M., Takeyama H., Tanaka T., Matsunaga T. (2004). Magnetic Cell Separation Using Antibody Binding With Protein A Expressed on Bacterial Magnetic Particles. *Analytical Chemistry*.

[44] Abts H., Emmerich M., Miltenyi S., Radbruch A., Tesch H. (1989). CD20 Positive Human B Lymphocytes Separated With the Magnetic Cell Sorter (MACS) Can Be Induced to Proliferation and Antibody Secretion In Vitro. *Journal of Immunological Methods*.

[45] Moore D. K., Motaung B., du Plessis N., Shabangu A. N., Loxton A. G. (2019). Isolation of B-Cells Using Miltenyi MACS Bead Isolation Kits. *PLoS One*.

[46] Helm M., Gollner K., Gollner U (2021). Isolation of Primary Human B Lymphocytes From Tonsils Compared to Blood as Alternative Source for Ex Vivo Application. *Journal of Chromatography B*.

[47] Abu-Reziq R., Alper H., Wang D., Post M. L. (2006). Metal Supported on Dendronized Magnetic Nanoparticles: Highly Selective Hydroformylation Catalysts. *Journal of the American Chemical Society*.

[48] Karimi Pasandideh E., Kakavandi B., Nasseri S (2016). Silica-Coated Magnetite Nanoparticles Core-Shell Spheres (Fe_3_O_4_@SiO_2_) for Natural Organic Matter Removal. *Journal of Environmental Health Science and Engineering*.

[49] Lu W., Ling M., Jia M., Huang P., Li C., Yan B. (2014). Facile Synthesis and Characterization of Polyethylenimine-Coated Fe_3_O_4_ Superparamagnetic Nanoparticles for Cancer Cell Separation. *Molecular Medicine Reports*.

[50] Mahdi Eshaghi M., Pourmadadi M., Rahdar A., Díez-Pascual A. M. (2022). Novel Carboxymethyl Cellulose-Based Hydrogel With Core–Shell Fe_3_O_4_@SiO_2_ Nanoparticles for Quercetin Delivery. *Materials*.

[51] Elmacı G. (2020). Magnetic Hollow Biocomposites Prepared from Lycopodium Clavatum Pollens as Efficient Recyclable Catalyst. *ChemistrySelect*.

[52] Abbas R. F., Hassan M. J. M., Rheima A. M. (2024). Adsorption of Fast Green Dye onto Fe_3_O_4_ MNPs and GO/Fe_3_O_4_ MNPs Synthesized by Photo-Irradiation Method: Isotherms, Thermodynamics, Kinetics, and Reuse Studies. *Sustainable Chemistry for the Environment*.

[53] Zulfiqar A. S., Afzal S., Khan R (2018). Structural, Optical, Dielectric and Magnetic Properties of PVP Coated Magnetite (Fe_3_O_4_) Nanoparticles. *Journal of Materials Science: Materials in Electronics*.

[54] Kanazaki K., Sano K., Makino A (2015). Development of Anti-HER2 Fragment Antibody Conjugated to Iron Oxide Nanoparticles for In Vivo HER2-Targeted Photoacoustic Tumor Imaging. *Nanomedicine: Nanotechnology, Biology and Medicine*.

[55] Cui Y. R., Hong C., Zhou Y.-L., Li Y., Gao X.-M., Zhang X.-X. (2011). Synthesis of Orientedly Bioconjugated Core/Shell Fe_3_O_4_@Au Magnetic Nanoparticles for Cell Separation. *Talanta*.

[56] Chellappa M., Vijayalakshmi U. (2019). Fabrication of Fe_3_O_4_-Silica Core-Shell Magnetic Nano-Particles and its Characterization for Biomedical Applications. *Materials Today: Proceedings*.

[57] Karimzadeh I., Aghazadeh M., Ganjali M. R., Norouzi P., Doroudi T., Kolivand P. H. (2017). Saccharide-Coated Superparamagnetic Fe_3_O_4_ Nanoparticles (SPIONs) for Biomedical Applications: An Efficient and Scalable Route for Preparation and In Situ Surface Coating Through Cathodic Electrochemical Deposition (CED). *Materials Letters*.

[58] Moradi S., Najjar R., Hamishehkar H., Lotfi A. (2022). Triple-Responsive Drug Nanocarrier: Magnetic Core-Shell Nanoparticles of Fe_3_O_4_@poly(N-Isopropylacrylamide)-Grafted-Chitosan, Synthesis and In Vitro Cytotoxicity Evaluation Against Human Lung and Breast Cancer Cells. *Journal of Drug Delivery Science and Technology*.

[59] Grüttner C., Müller K., Teller J., Westphal F., Foreman A., Ivkov R. (2007). Synthesis and Antibody Conjugation of Magnetic Nanoparticles With Improved Specific Power Absorption Rates for Alternating Magnetic Field Cancer Therapy. *Journal of Magnetism and Magnetic Materials*.

[60] Allard W. J., Matera J., Miller M. C (2004). Tumor Cells Circulate in the Peripheral Blood of All Major Carcinomas but Not in Healthy Subjects or Patients with Nonmalignant Diseases. *Clinical Cancer Research*.

[61] Silva J. G., Cárdenas R. A., Quiróz A. R (2014). Impedance Spectroscopy Assisted by Magnetic Nanoparticles as a Potential Biosensor Principle for Breast Cancer Cells in Suspension. *Physiological Measurement*.

[62] Vivek R., Thangam R., Kumar S. R (2016). HER2 Targeted Breast Cancer Therapy With Switchable “Off/On” Multifunctional “Smart” Magnetic Polymer Core–Shell Nanocomposites. *ACS Applied Materials and Interfaces*.

[63] Maltoni R., Gallerani G., Fici P., Rocca A., Fabbri F. (2016). CTCs in Early Breast Cancer: A Path Worth Taking. *Cancer Letters*.

[64] Choi W. I., Lee J. H., Kim J.-Y (2015). Targeted Antitumor Efficacy and Imaging via Multifunctional Nano-Carrier Conjugated With Anti-HER2 Trastuzumab. *Nanomedicine: Nanotechnology, Biology and Medicine*.

[65] Sun J., Zhou S., Hou P (2007). Synthesis and Characterization of Biocompatible Fe_3_O_4_ Nanoparticles. *Journal of Biomedical Materials Research Part A*.

[66] Calatayud M. P., Sanz B., Raffa V., Riggio C., Ibarra M. R., Goya G. F. (2014). The Effect of Surface Charge of Functionalized Fe_3_O_4_ Nanoparticles on Protein Adsorption and Cell Uptake. *Biomaterials*.

[67] Farjadian F., Ghasemi S., Mohammadi-Samani S. (2016). Hydroxyl-Modified Magnetite Nanoparticles as Novel Carrier for Delivery of Methotrexate. *International Journal of Pharmaceutics*.

